# Acute Kidney Injury Biomarker Responses to Short-Term Heat Acclimation

**DOI:** 10.3390/ijerph17041325

**Published:** 2020-02-19

**Authors:** Riana R. Pryor, J. Luke Pryor, Lesley W. Vandermark, Elizabeth L. Adams, Rachel M. Brodeur, Zachary J. Schlader, Lawrence E. Armstrong, Elaine C. Lee, Carl M. Maresh, Douglas J. Casa

**Affiliations:** 1 Center for Research and Education in Special Environments, Department of Exercise and Nutrition Sciences, University at Buffalo, SUNY, Buffalo, NY 14214, USA; lpryor@buffalo.edu; 2 Exercise Science Research Center, Department of Health, Human Performance and Recreation, University of Arkansas, Fayetteville, AR 72701, USA; lwvander@uark.edu; 3 Korey Stringer Institute, Department of Kinesiology, University of Connecticut, Storrs, CT 06269, USA; Elizabeth.adams@vcuhealth.org (E.L.A.); rachelvanscoy@gmail.com (R.M.B.); uconnla@aim.com (L.E.A.); Elaine.c.Lee@uconn.edu (E.C.L.); douglas.casa@uconn.edu (D.J.C.); 4 Department of Kinesiology, School of Public Health—Bloomington, Indiana University, Bloomington, IN 47405, USA; zschlade@indiana.edu; 5 Department of Human Sciences, The Ohio State University, Columbus, OH 43210, USA; maresh.15@osu.edu

**Keywords:** renal function, kidney injury, creatinine, heat stress, acclimation

## Abstract

The combination of hyperthermia, dehydration, and strenuous exercise can result in severe reductions in kidney function, potentially leading to acute kidney injury (AKI). We sought to determine whether six days of heat acclimation (HA) mitigates the rise in clinical biomarkers of AKI during strenuous exercise in the heat. Twenty men completed two consecutive 2 h bouts of high-intensity exercise in either hot (*n* = 12, 40 °C, 40% relative humidity) or mild (*n* = 8, 24 °C, 21% relative humidity) environments before (PreHA) and after (PostHA) 4 days of 90–120 min of exercise per day in a hot or mild environment. Increased clinical biomarkers of AKI (CLINICAL) was defined as a serum creatinine increase ≥0.3 mg·dL^−1^ or estimated glomerular filtration rate (eGFR) reduction >25%. Creatinine similarly increased in the hot environment PreHA (0.35 ± 0.23 mg·dL^−1^) and PostHA (0.39 ± 0.20 mg·dL^−1^), with greater increases than the mild environment at both time points (0.11 ± 0.07 mg·dL^−1^, 0.08 ± 0.06 mg·dL^−1^, *p* ≤ 0.001), respectively. CLINICAL occurred in the hot environment PreHA (*n* = 9, 75%), with fewer participants with CLINICAL PostHA (*n* = 7, 58%, *p* = 0.007), and no participants in the mild environment with CLINICAL at either time point. Percent change in plasma volume was predictive of changes in serum creatinine PostHA and percent changes in eGFR both PreHA and PostHA. HA did not mitigate reductions in eGFR nor increases in serum creatinine during high-intensity exercise in the heat, although the number of participants with CLINICAL was reduced PostHA.

## 1. Introduction

Hyperthermia, physical activity, and dehydration are commonly experienced by workers and athletes in hot environments [1,2,3,4,5], resulting in a transient impairment in kidney function as blood is redistributed from the kidneys to active musculature to maintain work intensity, and to the skin to thermoregulate [6]. While this reduction in renal blood flow may be benign, extreme hyperthermia, physical activity, and/or dehydration can lead to more severe reductions in renal blood flow, further reducing glomerular filtration rate (GFR), which is often manifested as reductions in creatinine clearance and a rise in serum creatinine [7]. In a clinical setting, the extent of the reductions of these biomarkers of kidney function is used to define acute kidney injury (AKI) [8,9]. Using these definitions, AKI is thought to occur in a variety of work and athletic settings conducted in hot environments, including the military [10], fire suppression [11], farming [12,13], American football [14], soccer [15], endurance running [16,17], and cycling [18]. Following a recovery period of 1–3 days, clinical biomarkers of AKI typically return to normal levels without lasting signs or symptoms of renal impairment [11,19]. However, epidemiological data demonstrate that nearly a third of individuals hospitalized with even mild AKI developed chronic kidney disease within one year, a rate much higher than those hospitalized without AKI [20].

Heat acclimation (HA) is a common strategy used to mitigate heat strain in physically active populations, resulting in adaptations such as improved heat dissipation, reduced perception of effort during activity, and greater physical performance [21,22,23,24,25]. HA may be a potential tool to maintain renal function during work in the heat due to hydration-related adaptations including increased renin and aldosterone, resulting in plasma volume expansion and increased sodium reabsorption, thereby reducing dehydration, hyperthermia, and possibly mitigating the reduction in renal blood flow [21,26]. Plasma volume expansion and other HA adaptations, such as enhanced sweat sensitivity and increased sweat rate reduce internal body temperature, thereby reducing heat strain [27,28,29,30]. Combined, these adaptations mitigate thermal and cardiovascular strain during physical activity and may have the potential to maintain kidney function and prevent kidney injury during heat strain, although this area of research is vastly unexplored.

Thus far, only three human studies have investigated the impact of HA on kidney function. First, an observational field study of collegiate American football players reported increases in clinical biomarkers of AKI over a 10 day HA period, with exercising serum creatinine increasing from the first to the tenth day of practice [14]. Over 43% of players were categorized as having Stage 1 AKI. However, percent change of GFR was calculated from values between days 0, 5, and 10, rather than within each day thereby limiting interpretation. Additionally, exercise intensity and duration were not reported and likely varied among days. Nevertheless, this was the first study to report changes in clinical biomarkers of AKI in healthy collegiate athletes throughout HA. Second, Omassoli et al. investigated changes in clinical biomarkers of AKI across a 23 days HA period [31]. HA reduced post-exercise serum creatinine and the number of individuals classified as having Stage I AKI during one hour of moderate intensity work in a warm environment. The third study involved military recruits who performed heavy intensity work during summer basic training, and reported no difference in serum creatinine increase during days 10 and 42 of training [10]. However, AKI was not quantified and pre-acclimatization values were not compared.

A study designed to bridge these important knowledge gaps is needed to understand the impact of HA on kidney function during high-intensity work in a hot environment, when risk for AKI is greatest. Determination and implementation of AKI prevention strategies would protect laborers from this health condition while likely also leading to employer cost savings. Therefore, the purpose of this study was to determine whether six days of HA mitigates changes in clinical biomarkers of AKI during strenuous work in a hot environment. We hypothesized that six days of HA would be protective against the reduction in kidney function during exercise heat stress.

## 2. Materials and Methods

### 2.1. Methods

Twenty healthy males free of cardiovascular, metabolic, respiratory, and renal diseases completed this laboratory, intervention study. An additional 20 males were either excluded due to meeting exclusionary criteria or withdrew from the study due to scheduling or unrelated personal reasons. Participants were not taking any medications known to affect thermoregulatory or cardiovascular variables. Participants began the study in a non-heat acclimated state and were tested from October to February in the northeastern United States where ambient temperature averaged 10 ± 2 °C and 70 ± 6% relative humidity (RH). All participants provided their informed consent prior to enrollment in the study, which was conducted in accordance with the Declaration of Helsinki. The protocol was approved by the (redacted for review) Institutional Review Board (H14-188). Upon study enrollment, participants were pair-matched by volume of aerobic exercise training prior to the study and maximal oxygen consumption (VO_2max_) and then randomized into one of two groups that exercised for six consecutive days in either a hot (40 °C, 40% RH) or mild (24 °C, 21% RH) room during all study visits.

Preliminary testing determined participant characteristics and study eligibility. Height to the nearest 0.5 cm and body mass to the nearest 0.02 kg were measured, as well as skinfold measurements at the chest, abdomen, and thigh in duplicate to calculate body fat percentage [32]. A graded exercise treadmill test and open circuit spirometry (TrueOne 2400 Metabolic Measurement System, Parvomedics, Sandy, UT, USA) measured VO_2max_. Participants with a VO_2max_ ≥ 45.0 mL·kg^−1^·min^−1^, an average maximal oxygen consumption for the young, male general population, were eligible to participate [33]. 

Laboratory visits occurred at the same time each day within subjects to account for the effect of circadian rhythm on thermoregulation. Participants refrained from alcohol, and strenuous exercise for 24 h, and caffeine for eight hours prior to all laboratory visits. Subjects drank 500 mL of water the night before and 250 mL of water the morning of each laboratory visit to ensure euhydration, which was confirmed with urine specific gravity (USG) via a refractometer (A300CL, Atago, Bellevue, WA, USA) upon arrival to the laboratory. Any subjects deemed hypohydrated (USG > 1.020) consumed 500 mL of water prior to proceeding with the protocol. Subjects inserted a rectal thermistor (401, Measurement Specialties, Beavercreek, OH, USA), 10–12 cm beyond the anal sphincter and wore a heart rate monitor (Race Trainer, Timex, Middlebury, CT, USA) throughout work.

### 2.2. Exercise Protocol

Participants completed a six-day exercise protocol in either a hot or mild environment. On days 1 and 6, participants were weighed, entered the exercise room, and rested quietly for 20 min. On each of these days, the exercise protocol was comprised of two, two-hour interval aerobic treadmill exercise sessions, separated by two hours of rest in a temperate environment. Intensities rotated among resting, walking at 4.83 km∙h^−1^, jogging at 60% VO_2max_, and running at 80% VO_2max_ to mimic intensities of Division I National Collegiate Athletic Association football preseason and are described elsewhere [34]. Subjects drank water ad libitum throughout all testing sessions, with the volume of water that participants consumed recorded. Exercise was terminated early if one of the following criteria was met: (1) T_re_ ≥ 40.0 °C, (2) unsteady gait making exercise unsafe, (3) signs or symptoms of heat illness, or (4) participant request. Exercising rating of perceived exertion was assessed using the validated OMNI 0–10 scale where 0 = “extremely easy” and 10 = “extremely hard” [35]. A 70 point environmental symptoms questionnaire was administered before and after each exercise session, with greater scores indicating more reported environmental symptoms and increased symptom severity, including dizziness, thirst, nausea, and trouble concentrating [36]. Before and after each exercise session, a blood draw was performed using an aseptic technique following 10 min of seated rest. During the two hours of rest, participants consumed a standardized meal. Upon the completion of the two exercise sessions USG was again assessed.

On day 2, participants completed a two-hour exercise session consisting of the same exercise intensities as days 1 and 6. This was completed as a part of a larger study, the results of which have been published previously [34]. On days 3–5, participants completed 90 min of cycling and/or treadmill exercise. Participants in the hot environment completed a hyperthermia-clamped protocol during which participants exercised at a self-chosen intensity to elevate rectal temperature to 38.5 °C during the first 30 min. The remaining 60 min consisted of maintaining rectal temperature between 38.5 °C and 39.9 °C through treadmill walking or cycling. Participants in the mild environment completed treadmill walking at 50% VO_2max_.

Following the blood draws, whole blood was allowed to clot—after which, samples were centrifuged at 3000 rpm for 15 min at 4 °C. Serum was transported to a commercial laboratory (Quest Diagnostics, Wallingford, CT, USA) to complete a comprehensive metabolic panel to quantify serum creatinine using isotope dilution mass spectrometry. Shifts in hemoglobin (HB 201+, Hemocue, Lake Forest, CA, USA) and hematocrit (model IEC MB centrifuge, Daemon/IEC Division, Needham Heights, MA, USA) measured in duplicate estimated percent change in plasma volume [37]. Cortisol concentration was measured by performing an ELISA in duplicate (CalBiotech, Spring Valley, CA, USA) with an intra-assay CV of ≤4.9%.

### 2.3. Data Management and Statistical Analysis

Increased clinical biomarkers of AKI (CLINICAL) were defined by two criteria: (1) the Kidney Disease Improving Global Outcomes criterion of an increase in serum creatinine ≥0.3 mg·dL^−1^ from pre-exercise [9] or (2) the Risk, Injury, Failure, Loss, Endstage Renal Disease criterion of a reduction in estimated glomerular filtration rate (eGFR) >25%, used in chronic kidney disease assessment [8]. These criteria were chosen due to their common use in clinical settings and use in similar previous research [31]. eGFR was calculated using the Chronic Kidney Disease Epidemiology Collaboration equation [38]. Changes in serum creatinine and percent changes in eGFR were calculated using measurements at baseline of the first exercise session and post-exercise of the second exercise session, separately on Days 1 and 6. Sweat rate was calculated from the change in body mass during exercise, accounting for fluid consumption and urinary losses. Area under the curve (AUC) for rectal temperature was calculated to determine the degree minutes above baseline rectal temperature each day using all time points (minutes 0, 15, 35, 55, 75, 95, 105, 115) during each exercise session, using a previously derived formula [39].

Between group (hot environment, mild environment) demographic characteristics were compared using independent t-tests. The two-way (group (hot environment, mild environment) by time (before heat acclimation (PreHA), after heat acclimation (PostHA)) mixed model ANOVA with post-hoc t-tests were performed to determine HA status. Evidence of HA is presented as mean differences (MD) and effect sizes (ES). ES was calculated using the Hedges’ g equation to determine the magnitude of differences between groups. Values of 0.2, 0.5, and 0.8 were considered small, medium, and large ES, respectively.

We explored the impact of HA on clinical biomarkers of AKI using two-way (group (hot environment, mild environment) by time (PreHA, PostHA)) mixed model ANOVA with post-hoc t-tests. Because we were interested in characterizing those who had increased clinical biomarkers of AKI (CLINICAL) and did not have increased clinical biomarkers of AKI (NO CLINICAL) during work in the heat, we stratified participants into these categories for further analyses. The incidence of CLINICAL was compared across time points (PreHA, PostHA) and groups (hot environment, mild environment) using chi-squared analyses and presented as percentages. CLINICAL and NO CLINICAL participants in the hot environment were compared to participants in the mild environment to explore differences in hydration, changes in clinical biomarkers of AKI, and physiological responses to exercise with separate one-way ANOVAs both PreHA and PostHA. To investigate the relationships among cardiovascular and thermoregulatory measures and hydration indices to changes in creatinine and percent changes in eGFR PreHA and PostHA, Pearson’s and Spearman’s Rho correlations were performed on eight cardiovascular, thermoregulatory, and hydration measures that would most likely predict kidney function (i.e., peak rectal temperature, rectal temperature area under the curve, percent change in plasma volume, total fluid consumed, percent fluid loss replaced, hematocrit, peak heart rate, and rating of perceived exertion). Stepwise linear regression analyses were performed on these variables to determine whether they could explain changes in creatinine or percent changes in eGFR. Statistical significance was set at *p* ≤ 0.05, adjusted with a Bonferroni correction when appropriate. Data are presented as the Mean ± SD. All statistical analyses were completed with SPSS version 21.0 (IBM Corp., Chicago, IL, USA).

## 3. Results

### 3.1. Heat Acclimation

Participants in the hot and mild environments had similar characteristics for all demographic variables (Mean ± SD; Age: 23 ± 4 years; Height: 179.3 ± 6.3 cm; Weight: 75.7 ± 7.3 kg; Body fat percentage: 11.2 ± 5.0%; VO_2max_: 53.0 ± 5.7 mL·kg^−1^·min^−1^; *p* > 0.05). Although not all indicators of HA reached statistical significance, the six-day protocol did elicit clinically relevant differences in the hot environment as indicated by the large mean differences and moderate to large ES for the following variables: end of exercise rectal temperature (Hot environment: MD: −0.41 ± 0.68 °C, ES = 0.77, *p* = 0.059; Mild environment: MD: −0.17 ± 0.30 °C, ES = 0.43, *p* = 0.152), peak heart rate (Hot environment: MD: −11 ± 7 bpm, ES = 1.36, *p* < 0.001; Mild environment: MD: −6 ± 11 bpm, ES = 0.51, *p* = 0.197), environmental symptoms (Hot environment: MD: −5 ± 7, ES = 0.55, *p* = 0.041; Mild environment: MD: −2 ± 2, ES = 0.42, *p* = 0.049), and perceived exertion (Hot environment: MD: −2 ± 2, ES = 0.59, *p* = 0.014; Mild environment: MD: −1 ± 1, ES = 0.25, *p* = 0.170).

### 3.2. Impact of HA on Clinical Biomarkers of AKI in HOT and MILD

**Figure 1:**
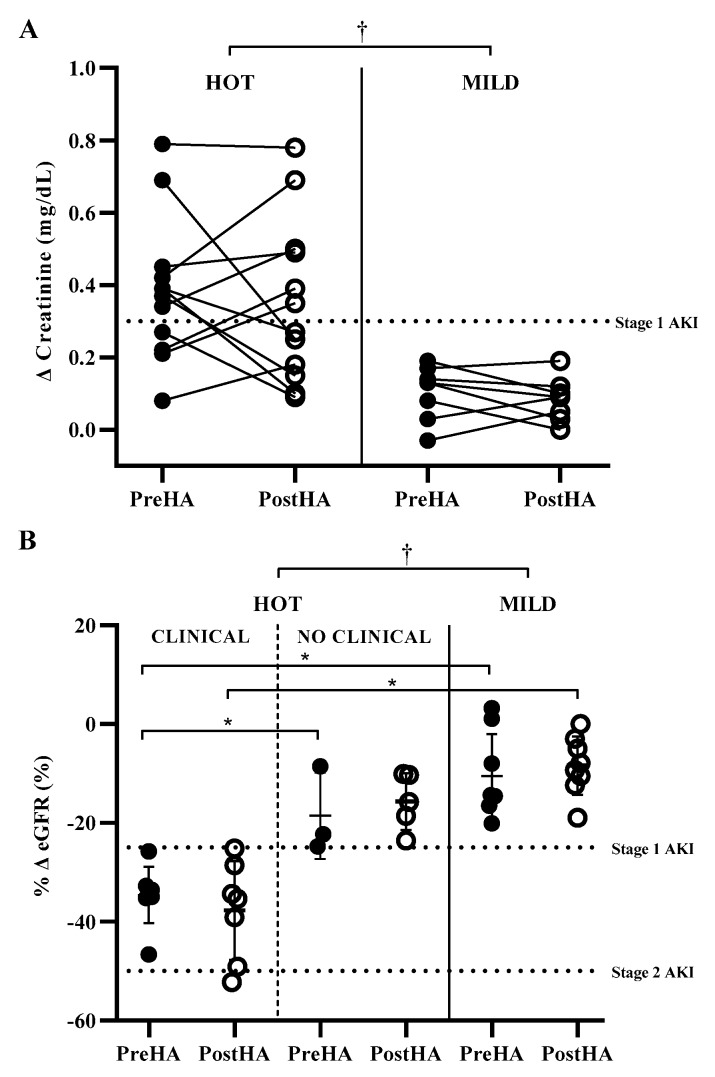
Clinical biomarkers of acute kidney injury (AKI) before (PreHA, closed circles) and after (PostHA, open circles) six days of heat acclimation. (**A**) Change in serum creatinine; (**B**) Percent change in estimated glomerular filtration rate (eGFR). Dashed horizontal lines indicate clinical biomarker AKI thresholds. * Difference between groups at the specified time point (*p* ≤ 0.05). † Main effect for group (*p* ≤ 0.05); HOT = hot environment; MILD = mild environment; CLINICAL = increased clinical biomarkers of acute kidney injury; NO CLINICAL = no increased clinical biomarkers of acute kidney injury.

Baseline serum creatinine was not different between participants in the hot environment PreHA (0.94 ± 0.09 mg·dL^−1^) and PostHA (0.96 ± 0.11 mg·dL^−1^, ES = 0.18, *p* = 0.723), with participants in the hot environment not different from participants in the mild environment (PreHA: 1.02 ± 0.10 mg·dL^−1^, ES = 0.77, *p* = 0.087; PostHA: 0.99 ± 0.08 mg·dL^−1^, ES = 0.28, *p* = 0.428), respectively. HA was not protective against elevations in clinical biomarkers of AKI. Changes in serum creatinine were not different in participants in the hot environment PreHA (0.39 ± 0.20 mg·dL^−1^) and PostHA (0.35 ± 0.23 mg·dL^−1^, ES = 0.17, *p* = 0.624), with participants in the hot environment greater than participants in the mild environment at each time point (PreHA: 0.11 ± 0.07 mg·dL^−1^, ES = 1.70, *p* ≤ 0.001; PostHA: 0.08 ± 0.06 mg·dL^−1^, ES = 1.46, *p* = 0.002) (Figure 1). Following the same pattern, percent change of eGFR in participants in the hot environment was not different PreHA (−30.2 ± 9.7%) and PostHA (−26.4 ± 12.4%, ES = 0.31, *p* = 0.395), with participants in the hot environment having greater reductions than participants in the mild environment at each time point (PreHA: −10.5 ± 8.5%, ES = 1.96, *p* ≤ 0.001; PostHA: −8.4 ± 5.9%, ES = 1.68, *p* ≤ 0.001).

### 3.3. Impact of Environment on CLINICAL Incidence

Clinical biomarkers of AKI only increased in participants exercising in the heat. PreHA, nine of the 12 (75%) participants in the hot environment and zero of the eight participants in the mild environment reached the threshold for Stage 1 AKI (χ^2^ (1) = 10.91, *p* ≤ 0.001). This indicates that environmental heat in addition to the heavy work intensity was necessary to increase clinical biomarkers of AKI.

### 3.4. CLINICAL, NO CLINICAL, and Mild Environment PreHA

We explored differences in heat strain and hydration between CLINICAL participants, NO CLINICAL participants, and participants in the mild environment. By definition, CLINICAL PreHA had a greater change in creatinine and percent change in eGFR (0.46 ± 0.17 mg·dL^−1^; −34.6 ± 5.7%) than NO CLINICAL (0.17 ± 0.08 mg·dL^−1^, ES = 1.56, *p* = 0.010; −18.6 ± 8.7%, ES = 2.09, *p* = 0.017), respectively. CLINICAL also had greater changes than participants in the mild environment (0.11 ± 0.07 mg·dL^−1^, ES = 2.61, *p* ≤ 0.001; −10.5 ± 8.5%, ES = 3.00, *p* ≤ 0.001), respectively. Change in creatinine and percent change in eGFR were similar between NO CLINICAL and participants in the mild environment (ES = 0.68, *p* = 0.741; ES = 0.78, *p* = 0.275), respectively, although moderate-to-large effect sizes were measured. There was a large effect size for rectal temperature between CLINICAL (39.73 ± 0.42 °C) and NO CLINICAL (39.13 ± 0.35 °C, ES = 1.23, *p* = 0.059), although this did not reach statistical significance. Rectal temperature of participants in the mild environment (38.20 ± 0.30 °C) was lower than both CLINICAL (ES = 3.73, *p* ≤ 0.001) and NO CLINICAL (ES = 2.37, *p* = 0.005). Cortisol fold change was similar among CLINICAL (2.3 ± 1.2), NO CLINICAL (2.1 ± 0.2), and participants in the mild environment (1.6 ± 0.5, *p* = 0.597). Differences in hydration indices are reported in Table 1.

### 3.5. CLINICAL, NO CLINICAL, and Mild Environment PostHA

Following six days of exercise in a hot or mild environment, seven of the 12 (58%) participants in the hot environment participants and zero participants participants in the mild environment were classified as CLINICAL (χ^2^ (1) = 7.18, *p* = 0.007). By definition, CLINICAL PostHA had a greater change in creatinine (0.50 ± 0.18 mg·dL^−1^) than NO CLINICAL (0.15 ± 0.07 mg·dL^−1^, ES = 2.01, *p* ≤ 0.001) and was also greater than participants in the mild environment (0.08 ± 0.06 mg·dL^−1^, ES = 2.84, *p* ≤ 0.001), with similar changes between NO CLINICAL and participants in the mild environment (ES = 0.94, *p* = 0.568). PostHA percent change in eGFR was greater in CLINICAL (−24.8 ± 10.7%) than MILD (−5.6 ± 5.9%, ES = 2.00, *p* ≤ 0.001), but was similar to NO CLINICAL (−12.6 ± 8.4%, ES = 1.04, *p* = 0.059), which was also similar to participants in the mild environment (ES = 0.87, *p* = 0.347). PostHA rectal temperature was similar between CLINICAL (39.39 ± 0.70 °C) and NO CLINICAL (39.35 ± 0.47 °C, ES = 0.06, *p* = 1.000), with both groups having a greater rectal temperature than participants in the mild environment (38.04 ± 0.49 °C; ES = 1.99, *p* ≤ 0.001; ES = 2.35, *p* = 0.002), respectively. There were no differences in PostHA cortisol fold change among CLINICAL (1.2 ± 0.9), NO CLINICAL (2.2 ± 1.1), and participants in the mild environment (1.2 ± 1.0, *p* = 0.231).

### 3.6. Relationships among Clinical Biomarkers of AKI and Cardiovascular Strain, Thermoregulatory Strain, and Hydration

We conducted stepwise linear regressions with Pearson’s and Spearman’s Rho correlations to determine the relationships among cardiovascular strain, thermoregulatory strain, and hydration indices and change in creatinine and percent change in eGFR in participants in the hot environment PreHA and PostHA. PreHA, percent fluid replaced and percent change in plasma volume had the strongest relationships with change in creatinine. However, no variables were significant predictors of change in creatinine. Percent change in plasma volume had the strongest relationship and was the only significant predictor of percent change in eGFR PreHA (r = 0.64, *p* = 0.033). PostHA, percent change in plasma volume had the strongest correlations and was the only significant predictor of both change in creatinine (r = −0.73, *p* = 0.007) and percent change in eGFR (r = 0.72, *p* = 0.008).

## 4. Discussion

This is the first study to assess the impact of HA on changes in clinical biomarkers of AKI during high-intensity exercise in a hot environment. We found that six days of HA was not protective against the reduction in kidney function when exercising in the heat, although the number of people reaching the clinical biomarker threshold for AKI was reduced following HA. This suggests that HA may reduce the risk of developing AKI during high-intensity exercise in the heat, although renal function remains impaired. Our findings in combination with the findings of others support the notion that HA can mitigate decrements in renal function in individuals who work in hot environments, such as agricultural workers, military members, and athletes. While this topic has only been explored in a few studies, efforts should be continued to determine mechanistic explanations of renal function impairment and preservation during exercise in the heat.

Renal blood flow is reduced at rest in a hot compared to a cool environment, with further reductions during exercise in the heat, although this response is normal and considered to be benign [6,7]. GFR is preserved during light and moderate intensity work in a cool environment, with a 15–19% reduction in GFR during moderate work in the heat [6,7]. The addition of dehydration to exercise in the heat further decreases GFR by up to 51% [7], surpassing the clinical biomarker threshold of a GFR reduction of >25% in chronic kidney disease assessment [8], thereby possibly indicating increased risk of developing AKI. The current study corroborates these observations, with an eGFR reduction of 30% following four hours of heavy intensity work in a hot environment PreHA, classifying over half of the participants as potentially having AKI. Reduction in GFR leads to a reduction in creatinine clearance, resulting in an accumulation of serum creatinine, evidenced by a 0.39 mg·dL^−1^ increase in creatinine in our participants. The eGFR reduction and creatinine increase observed in HOT mirrors changes in previous studies in soccer, downhill running, and military basic training in the heat [10,15,40].

Omassoli et al. studied the impact of HA on clinical biomarkers of AKI during moderate intensity work in 27 °C Wet Bulb Globe Temperature, a slightly cooler environment than the current study [31]. Both studies used a duration of six days of HA to compare renal responses and found nearly identical responses of GFR and creatinine PreHA. However, following six days of HA, the previous participants experienced more preserved renal function including mitigated GFR reduction and creatinine increase than the current study, leading to a reduced number of participants reaching the clinical biomarker threshold for AKI PostHA. The two HA and testing protocols varied drastically, making comparisons between studies complex. Their HA protocol consisted of 100 min walking in a warm environment while we used a hyperthermia-clamped protocol and tested at a greater exercise intensity in a much hotter environment. Despite any renal function benefits potentially gained during HA and possible reduced risk of AKI, the oppressive testing environment and high-intensity work in the current study, which is known to lead to exacerbated heat strain on the second day of two consecutive days of work [34,41], may have overshadowed these benefits. Additionally, a more traditional HA period of 10–14 days may be needed to mitigate the rise in clinical biomarkers of AKI and reduce AKI risk.

Mechanisms of enhanced renal function following HA have been proposed but not directly studied. Plasma volume can expand by 3–27% following HA, and generally occurs during days 3–6 of HA [21,42]. This cardiovascular variable provides a thermoregulatory benefit of improved heat dissipation by increasing sweat volume to enhance evaporative cooling, thus mitigating core body temperature rise [43]. Plasma volume expansion may preserve renal function by maintaining cardiac output, mitigating the reduction in renal blood flow during heat stress through the renin-angiotensin system, permitting a greater absolute reduction in plasma volume before vasoconstriction of the renal arterioles. Additionally, sympathetic activation [25,44], which causes reduced blood flow to the kidneys [6], is reduced following HA. This lends credence to HA reducing the risk of AKI, although this hypothesis remains untested. Plasma volume expansion and reduced sympathetic activation were likely both present in this study, as six days of HA falls within the time course of these adaptations, although they were not directly measured. In contrast, studies in rats and hamsters indicate reduced renal function following three to ten weeks of passive HA in warm environments, causing reduced renal blood flow and GFR [45,46]. Thus far, no human studies have reported responses of renal blood flow and GFR to HA.

Only participants who exercised in the heat reached the clinical biomarker threshold for AKI, both PreHA and PostHA. Participants in the hot environment achieved a greater rectal temperature than participants in the mild environment due to the thermally stressful environment and had a greater fluid turnover, indicated by greater sweat losses and water consumption, although both groups ended exercise with a similar body mass loss, indicative of similar percent dehydration at the end of exercise. Although not measured in the current study, participants in the hot environment likely experienced an approximately one liter per minute reduction in cardiac output as compared to participants in the mild environment, similar to a previous study that implemented moderate-high-intensity exercise in similar ambient conditions [47]. It is possible that CLINICAL had further reduced cardiac output than NO CLINICAL, resulting in further reduced renal perfusion and renal function, indicated by greater changes in serum creatinine and eGFR. This theory is supported by our data, with greater reductions in plasma volume being predictive of greater rises in serum creatinine and greater reductions in eGFR. Our findings suggest that mitigating plasma volume reductions during work in the heat may play a key role in maintaining renal function, thereby preventing AKI. In practice, individuals exercising in the heat should drink water in a manner that prevents dehydration, by either drinking to thirst or drinking according to body mass loss when available, thereby mitigating plasma volume reductions [48].

Omassoli et al. suggested that limiting work intensity based on ratings of perceived exertion may be useful to prevent AKI [31]. However, our results indicate similar perceived exertion among individuals who met and did not meet the clinical biomarkers thresholds for AKI. This difference in findings may relate to our more intense testing protocol resulting in greater exertion and a smaller range of responses than the previous study, with all subjects reporting at least a 4/10 on our 0–10 perceived exertion scale at the end of exercise, compared to subjects in the previous work reporting a range from the lowest to the highest possible values for exertion (6–20 on the Borg rating of perceived exertion scale) [49]. It is possible that perceived exertion may be predictive during lower-intensity work, but it should be considered that AKI is more likely to occur during higher intensity work such as our protocol in which perceived exertion was not predictive of clinical biomarkers of AKI [40,50,51].

Similar to previous studies, we measured serum creatinine and eGFR and used these biomarkers of kidney function to classify individuals as having or not having AKI using the Kidney Disease Improving Global Outcomes criterion of an increase in serum creatinine ≥0.3 mg·dL^−1^ from pre-exercise [9] and the Risk, Injury, Failure, Loss, Endstage Renal Disease criterion of a reduction in eGFR >25%. It should be emphasized that, while serum creatinine is a biomarker of AKI, eGFR may not be valid in the acute setting, but instead is a valid measure of chronic kidney disease. Additionally, we did not confirm the presence of AKI. There are over 100 studied biomarkers of AKI, with neutrophil gelatinase-associated lipocalin (NGAL), tissue inhibitor of metalloproteinase 2 (TIMP-2), and insulin-like growth factor binding protein 7 (IGFBP7) having been studied in laboratory-based human subjects testing. NGAL is the most widely studied biomarker of AKI which is upregulated and released into circulation in response to renal tubular injury, and is commonly used in the clinical setting as an early biomarker of subclinical AKI to determine prognosis [52,53,54,55] and AKI severity [56,57,58]. More recently, it has been studied as a tool to determine AKI severity during heat exposures with promising results [11,18,19]. Urinary TIMP-2 and IGFBP7 are biomarkers recently approved by the Federal Drug Administration that successfully indicate future risk of AKI in hospitalized individuals [59,60,61], with the usefulness of these markers in predicting AKI in healthy individuals during heat stress only now beginning to be understood [62]. Therefore, future research should determine the utility of these biomarkers in predicting future risk of AKI in individuals during acute heat exposures and throughout HA.

Future research should determine AKI risk in females during work in a hot environment. There are a variety of protocols that induce HA using varying stressors such as the traditional constant work rate protocol, as well as sustained forcing function based on relative intensity such as hyperthermia-clamped or heart rate-clamped protocols [63]. The impact of HA protocols on AKI risk warrants further investigation.

## 5. Conclusions

With the high number of individuals partaking in physically demanding activities in hot environments, the potential for AKI in this population is high, generating a need to create effective AKI prevention strategies. We demonstrated that six days of HA did not mitigate the rise in serum creatinine during exercise in the heat, although the number of participants classified as CLINICAL was reduced PostHA compared to PreHA. Hydration indices (Table 1) had the greatest correlation to increased serum creatinine during work in the heat, providing a starting point for further work to establish useful AKI prevention and intervention strategies. Additionally, determining the true incidence of AKI during physical tasks in the heat and throughout HA is warranted.

## Figures and Tables

**Table 1:** Hydration markers among participants with and without increases in clinical biomarkers of acute kidney injury before and after six days of heat acclimation.

Measurement	PreHA				PostHA			
	Hot		Mild (*n* = 8)	ES	Hot		Mild (*n* = 8)	ES
	CLINICAL (*n* = 9)	NO CLINICAL (*n* = 3)			CLINICAL (*n* = 7)	NO CLINICAL (*n* = 5)		
Urine Specific Gravity	1.018 ± 0.009	1.012 ± 0.008	1.014 ± 0.007	0.57	1.018 ± 0.008	1.022 ± 0.010	1.018 ± 0.009	0.01
Sweat Rate (L·h^−1^)	1.70 ± 0.37 *	1.44 ± 0.12 *	0.76 ± 0.08	0.65	1.86 ± 0.57	2.04 ± 0.43 *	1.05 ± 0.80	0.29
Total Sweat Loss (L)	5.81 ± 1.23 *	5.29 ± 0.97 *	2.65 ± 0.52	0.37	6.31 ± 1.22 *	5.51 ± 0.85 *	2.87 ± 0.79	0.62
Fluid Consumption (L)	3.61 ± 1.33 *	4.79 ± 2.13 *	1.79 ± 0.95	0.65	4.76 ± 1.82 *	3.39 ± 1.00	1.68 ± 0.83	0.74
Percent Fluid Replaced (%)	61.9 ± 18.9	88.6 ± 25.6	70.6 ± 40.5	1.10	73.2 ± 16.4	61.9 ± 16.3	60.5 ± 33.3	0.58
Percent Δ Plasma Volume (%)	−9.0 ± 3.9 *	−3.7 ± 5.6	−0.6 ± 5.8	1.04	−7.1 ± 5.4	−3.5 ± 3.3	−1.3 ± 4.5	0.65

PreHA = before heat acclimation; PostHA = after heat acclimation; Hot = hot environment; Mild = mild environment; CLINICAL = increased clinical biomarkers of acute kidney injury; NO CLINICAL = no increased clinical biomarkers of acute kidney injury; ES = effect size of CLINICAL and NO CLINICAL group. * Different than MILD at the specified time point (*p* < 0.05).
